# Musculoskeletal Consequences in Cyber-Addicted Students - Is It Really A Matter of Health? A ROC Curve Analysis for Prioritizing Risk Factors

**DOI:** 10.34172/jrhs.2020.10

**Published:** 2020-04-21

**Authors:** Rashid Heidarimoghadam, Alireza Mortezapour, Fakhradin Ghasemi, Mohammad Ebrahim Ghaffari, Mohammad Babamiri, Mahdi Razie, Khadijeh Bandehelahi

**Affiliations:** ^1^Research Center for Health Sciences, Hamadan University of Medical Sciences, Hamadan, Iran; ^2^Department of Ergonomics, School of Public Health, Hamadan University of Medical Sciences, Hamadan, Iran; ^3^Occupational Health and Safety Research Center, Hamadan University of Medical Sciences, Hamadan, Iran; ^4^Dental Sciences Research Center, School of Dentistry, Guilan University of Medical Sciences, Rasht, Iran; ^5^Social determinants of Health Research Center, Hamadan University of Medical Sciences, Hamadan, Iran; ^6^Department of Public Health, Asadabad School of Medical Sciences, Asadabad, Iran

**Keywords:** Neck Pain, Behavior, Addictive, Social media, Video Games, Addiction Medicine

## Abstract

**Background:** The high prevalence and also multiple consequences of addiction to various online content, including online games and social networks, have become a major challenge. The ability to predict musculoskeletal disorders from this addiction can help reveal in students' health status in the near future. The aim of this study was to determine the prevalence of this addiction and the ability to predict neck pain from this matter in students.

**Study Design:** A cross sectional study.

**Methods:** This study was carried out among 665 students. Data collection was performed through three questionnaires on problematic use of online games, social networking addiction, and Nordic musculoskeletal disorders. Data were compared with Chi-square and independent T-test, and the logistic regression model was then presented at a significant level of 0.05. Finally, the area under the receiver operating characteristic (ROC) curve and Discriminant analysis were conducted to clarify associations.

**Results:** The prevalence of Internet-related content addiction was 32.8%. The results showed that addiction to social networks and online games can increase the risk of neck disorder. Also 0.58 area under ROC curve depicted the ability to predict neck pain from this addiction.

**Conclusions:** In students with internet - related content addiction, neck disorder can be predicted. Given the high prevalence of internet addiction in students, it is essential to take immediate and appropriate interventions to avoid the associated adverse effects such as neck problems.

## Introduction

Today, in all societies, students are recognized as an important group^1^. Around one-half of US 18 to 24 years citizen are enrolled in universities ^2^. Right now, more than four million students study at various universities in Iran^3^. Attending students in universities have many ways through which they might have a promotive effect on mental health^1^. Attending the university can be a ground for students to grow and develop, but it can also provide a variety of psychological disorders^4^. Adolescents and youth are more susceptible to psychological abnormalities due to the rapid age-related changes^5^. Diminution of immunity to the suffering and impairment associated with mental illness is one of their attributes^1^. Students represent a substantial subject in which to access to mental health care^6^. Mental health problems are highly prevalent among university students and it has caused concern^1^. The last decade has witnessed an enormous increase in the body of literature on the newly emerging mental health issues associated with Internet addiction in students^7^. Internet addiction is not yet a DSM IV diagnosis, but its definition is based on the DSM IV addictive behavior criteria^8^. Among university students, internet addiction prevalence has been estimated to 2.8% in Iran^9^. Various problematic use of the internet has been reported among university students^10^. Gaming has been much investigated as an online application with an incredibly high addictive output^11^. Moreover, the use of social networks is another one^12^. Universities have started to consider the Internet dependence issue and ways through which it might influence their students^13^. Problematic Internet use interrupts main function of the musculoskeletal system^14^**(17)**. Social network addiction and psychosomatic outcomes were subjects of a comprehensive systematic review^15^. Adverse Health Effects (neck and shoulder pain) were demonstrated to be a challenging issue among Medical Students who use Facebook addictively^16^. The relationship between online gaming addiction and Korean students' carpal tunnel syndrome was the subject of a recent study^17^. Zheng et al. showed that Overall prevalence of cervical pain in problematic internet user was 48%^14^. One of the most remarkable health challenges in students is musculoskeletal disorders, especially in the neck^18^. A prospective cohort study showed that Neck pain is quite common among students^19^. Musculoskeletal disorders were most commonly declared by students at their neck^20^. In previous studies, there have been mentioned many reasons for neck disorder^21^. From all, psychosocial factors were significant predictors for such a problem ^22^. The issue of Internet addiction a psychosocial issue - ^23^and its association with musculoskeletal disorders in the neck has received less attention. So the aim of present study was to investigate the relationship between cyber addiction and neck disorder and compare the importance of independent variables among university students.

## Methods

This cross-sectional study was conducted at Hamadan university of Medical Sciences in 2018. The sample was randomly selected from the population of students. After receiving the goals and accepting the informed consent form, all participants in this study (n=665) were entered into the study. All the scientific and executive stages of this study have been approved by the Ethics Committee (registry number: UMSHA.REC.1397.314). The completion of at least one year of university education was the main criterion for inclusion in this study. Students with previous history of neck injury or disorder (as a self - reported question), were excluded from the study. The following questionnaires were used for data collection. Demographic information was also collected.

### Virtual Social Network Addiction Questionnaire

Problematic use of social media, also known as social media addiction, is a proposed form of psychological or behavioral dependency, such as gaming disorder, Internet addiction disorder, and other forms of digital media overuse. In the Currebt et al. study virtual social network addiction questionnaire was used. This tool contains 43 questions that were rated as five-point Likert (never, almost never, sometimes, often and always). Seven dimensions of addiction to virtual social networking are measured in this questionnaire. These dimensions include loss of control, academic failure, dependency, avoidance syndrome, mood changes, reduced interest in other activities and social conflicts. The minimum and maximum attainable scores for each student were 43 and 215, respectively. To answer the main questions of the study, only the total score of the questionnaire, which represents the total score of social network addiction, was used. The validity and reliability of this questionnaire were approved in the Lindsay et al. study^24^. This tool also used in Iranian students after the acquisition of an acceptable level of reliability and validity in the same culture study ^25^.

### Problematic online Game Questionnaire

The growing popularity of online games has led to the introduction of excessive gaming that in some cases can lead to psychological problems. In this study we used one of the well-known instruments regards to assess the dependency of students to online games. The instrument has 18 questions to be answered by the participants in the five - point Likert (never, rarely, sometimes, often, and always). This tool has six sub-scales, including social isolation, interpersonal conflicts, excessive use, neglect, immersion and fascination. In order to measure addiction to online games, only the final scores achieved were used in the statistical analysis. Aminimanesh et al. ^26^ have validated this instrument in accordance with Iranian Culture (Cronbachs Alpha was 0.86).

### Nordic musculoskeletal questionnaire

Data on neck musculoskeletal disorders were collected by Nordic questionnaire. Participants answered questions on neck disorder in three periods, in general, in the past month and the day before. Their responses were based on a body diagram.

In this study, descriptive results were reported in terms of mean, percentage and standard deviation. Participants' data were compared using Chi-square, T-test and then simple and multiple logistic regression were presented. The surface under the receiver operating characteristic (ROC) curve was used to compare the importance of independent variables in prediction neck pain. Discriminant analysis was utilized to propose a function to classify people who were not involved in the analysis into one of the two groups - with and without neck pain-. The level of confidence in presenting results was 95 percent. The SPSS version 16 and Stata version 11 software packages were used for data analysis.

## Results

Out of 665 subjects, 250 (37.6%) were male and 415 (62.4%) were female. Based on the level of education, Bachelor degree with 350 students (52.6%) and associate degree with 60 students (9%) were the highest and lowest number of participants in the study (Table 1). The mean (standard deviation) of the students' age was 22.12(0.15) years (Table 2). Prevalence of addiction to Internet-related contents (students who were simultaneously addicted to online games and social networks) was 32.8 percent among participants.

**Table 1:** The frequency of subjects by gender and level of education in the two study groups

**Variables**	**Neck pain**		***P*** **value**
	**No (%)**	**Yes (%)**	
Gender			0.020
Male	97 (38.8)	153 (61.2)	
Female	126 (30.4)	289 (69.6)	
Education			0.080
Associated degree	28 (46.7)	32 (53.3)	
Bachelor degree	109 (31.1)	241 (68.9)	
MSc degree	36 (37.5)	60 (62.5)	
PhD	50 (31.4)	109 (68.9)	

**Table 2:** Effect of independent variables on neck pain using logistic regression

**Variables**	**Odd ratio (95% CI)**	***P*** **value**	**Adjusted Odd ratio (95% CI)**	***P*** **value**
Associated degree	1.00	0.093	1.00	0.239
Bachelor degree	1.93 (1.11, 3.37)	0.020	1.57 (0.88, 2.78)	0.123
MSc degree	1.46 (0.76, 2.80)	0.258	1.37 (0.69, 2.72)	0.360
PhD	1.91 (1.04, 3.50)	0.037	1.89 (1.01, 3.55)	0.047
Female	1.45 (1.05, 2.02)	0.026	1.83 (1.28, 2.62)	0.001
Age	0.99 (0.96, 1.04)	0.884	0.99 (0.95, 1.04)	0.960
Cyber Addiction	2.28 (1.57, 3.31)	0.001	2.45 (1.65, 3.63)	0.010

The individual and simultaneous (adjusted) effects of each independent variable are shown in the above-mentioned table. The unadjusted effect of the independent variables showed that the level of education, gender and Internet-related content addiction significantly affected the neck pain. The probability of developing neck pain in bachelor and Ph.D. students was 1.57 and 1.89 times higher than associate students. The chance for women to develop neck pain was 1.83 against men. The odds ratio of Internet - addicted students to develop neck pain compared to non-addicted students was 2.45.

The effect of each independent variable on neck disorder with the ROC curve analysis showed significant differences (p=0.048). The most significant variables were internet addiction, gender, level of education and age, respectively (Table 3).

**Table 3:** Compare the importance of each independent variables on the response variable using the ROC curve

**Variables**	**Number**	**ROC area (95% CI)**	***P*** **value**
Age	665	0.51 (0.47, 0.56)	0.04
Gender	665	0.54 (0.50, 0.58)	
Education	665	0.52 (0.48, 0.56)	
Cyber Addiction	665	0.58 (0.54, 0.62)	

**Figure 1:**
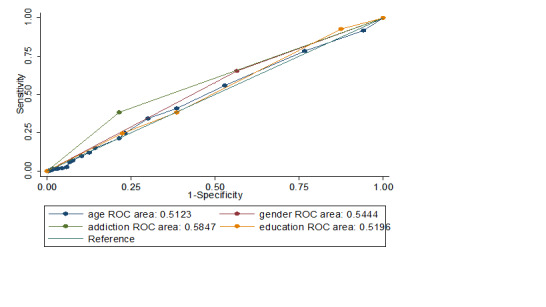
Surface area below the curve for each variable

Figure 1 shows the area below the curve for each of the variables. The values below the curve indicate the importance of different variables in classifying students with or without neck pain. The values below the curve for each independent variable demonstrated the highest predictive accuracy of the Internet addiction. It can therefore be concluded that Internet addiction has the highest accuracy and thus the main priority in predicting neck pain in students. Gender, education and age were then prioritized. Chi-square test was used to compare Internet addiction with other three independent variables and the results are presented in Table 4.

**Table 4:** Comparing each of independent variables with Internet addiction

**Variables**	**Number**	**Chi2**	***P*** **value**
Gender	665	1.96	0.162
Age	665	6.28	0.012
Education	665	4.99	0.020

Comparison of independent variables with internet addiction-most important-showed that age (p=0.012) and education (p=0.025) differed significantly, while gender (p=0.162) has not. It means that addiction and gender have the same effect. Function 1 was obtained using discriminant analysis to predict the chance of neck pain for students.

Y= -3.087 + 0.30 (*Education* ) + 1.30 (*Gender* ) – 0.02 (*Age* ) + 1.95 (*Addiction* )

Function 1: the prediction function of individuals who can be placed in the neck pain group. This function can be used to classify students to a group that could have neck pain.

## Discussion

The purpose of this study was to musculoskeletal pain in the neck through cyber addiction in the form of virtual social networks and online games. The results showed that addiction could increase the chance of neck disorder by 245%. Along with the results of this study, Zheng et al. showed that over use of the Internet and its contents could cause pain in the neck^14^. In line with the results of the present study, another survey showed that addiction to virtual social networks can cause discomfort in the students' neck^27^. The results of the review studies, on the other hand, confirmed this study so that problematic Internet gaming can lead to psychosomatic disorders, including neck pain^28^. In addition to the inappropriate positions that people take when using mobile phones and computers; Internet addiction causes fatigue, lack of energy, and sleep problems, all of them are associated with musculoskeletal disorders^29^. However, in contrast to the results of this study, the results of some studies indicated that there was no relationship between Internet- related content addiction and neck pain^30^. Maybe the difference between socio- economic status and different cultural factors in communities can be interpreted. In other words, these differences can arise at the train level for people regarding emerging issues such as Internet addiction^31^.

Other results showed that the risk of neck disorder in women can be higher. Stock et al. studied students from seven different countries and confirmed the results of this study that women are more likely to develop musculoskeletal disorders in the neck^32^. In line with the results of this study, Dianat et al. highlighted the difference between male and female students in the report of neck pain^33^. In explaining the impact of gender, it can certainly be concluded that female gender has been considered to be a risk factor for neck pain. This is because of differences in women's and men's musculoskeletal systems. Readiness to report or seek medical care for pain or discomfort can be another factor.

Students with higher education were more likely to have neck disorder in this study. Contrary to the results of this study, some studies showed that the level of education has no significant effect on the incidence of musculoskeletal disorders in the neck and shoulders^34^. In a study in Finland, it was found that the risk of pain in the neck region was reduced with increasing levels of education^35^. Perhaps the reason for this difference is the difference in the studied population or the amount of time a student uses the internet-related content. In line with the results of this study, Strine et al. studied 29,828 American adults and reported that neck pain was higher in the subjects with higher education^36^. In general, few studies have confirmed that an increase in education can be a risk factor for incidence of neck disorder. This could perhaps be attributed to the time spent on portable computers and touchscreen telephones in higher educated students accessing online content such as research - based activities. The results of multiple logistic regression showed that the students' age did not significantly affect the neck disorder. Other studies in students and workforces also reported results similar to the present study ^37^. In general, increase in age has always been recognized as a significant risk factor for musculoskeletal disorders in previous studies^38^. This contradiction in results can be attributed to the narrow age range in this study.

Other results showed that addiction to Internet-related content (including addiction to online games and virtual social networks), gender and education are the most important predictors of neck disorder.

Considering the novelty of this study, the results showed that rock could be used to identify the most important predictions of a variable and estimate their effects. In this study, the addiction to Internet-related content, gender, and education level in the prediction of neck pain was prioritized respectively. There has been no study in the past that prioritizes the predictors of neck pain in students. However, it can be concluded from previous studies that the effect size or odds ratio of different variables in the prediction of neck pain is not the same. In a 1-year longitudinal study Leclerc et al. showed that psychological distress and psychosomatic problems had a higher odds ratio than gender for occurrence of neck pain in the next one year^39^. Carroll et al. mentioned that psychosocial factors have a higher predictive power than other factors (individual, occupational, etc.) for neck pain^40^. Despite the differences between the odds ratio, its significance cannot be clearly recognized. Hence, Using ROC regression to prioritize variables is recommended.This study, likewise other cross-sectional ones, cannot determine causal relationship because of not having time dimension. It is suggested that the efficacy of ROC model being tested in longitudinal studies.

## Conclusion

According to the results, the ability to predict neck pain from addiction to various internet-related content was approved. Due to the relatively high prevalence of this addiction in students, there is a necessity to plan and perform appropriate interventions based on importance of predictors to reduce adverse effects, such as neck pain.

## Acknowledgements

We appreciate help of Saeedeh Mosaferchi – MSc of ergonomics – for her review of the manuscript. Authors herby thankful of students for their contribution in this study.

## Conflict of interest

The authors declare that they have no competing interests

## Funding

Funding for this study was provided by The Vice-Chancellor of Research and Technology of Hamadan university of Medical sciences Grant 9705092792. This founder had no role in the study design, collection, analysis or interpretation of the data, writing the manuscript, or the decision to submit the paper for publication.

## Highlights

- This study depicted the relationship between internet addiction and MSDs
- The application of ROC was presented for internet addiction researches
- Addiction to internet has significantly more effect in prevalence of MSDs in comparison to other studied variables
